# Perturbational blowup solutions to the compressible Euler equations with damping

**DOI:** 10.1186/s40064-016-1766-8

**Published:** 2016-02-27

**Authors:** Ka Luen Cheung

**Affiliations:** Department of Mathematics and Information Technology, The Hong Kong Institute of Education, 10 Lo Ping Road, Tai Po, New Territories Hong Kong

**Keywords:** Blowup, Global existence, Euler equations, Perturbational method, Damping, Singularity, 35Q53, 35B44, 35C05, 35C06

## Abstract

**Background:**

The *N*-dimensional isentropic compressible Euler system with a damping term is one of the most fundamental equations in fluid dynamics. Since it does not have a general solution in a closed form for arbitrary well-posed initial value problems. Constructing exact solutions to the system is a useful way to obtain important information on the properties of its solutions.

**Method:**

In this article, we construct two families of exact solutions for the one-dimensional isentropic compressible Euler equations with damping by the perturbational method. The two families of exact solutions found include the cases $\gamma >1$ and $\gamma =1$, where $\gamma$ is the adiabatic constant.

**Results:**

With analysis of the key ordinary differential equation, we show that the classes of solutions include both blowup type and global existence type when the parameters are suitably chosen. Moreover, in the blowup cases, we show that the singularities are of essential type in the sense that they cannot be smoothed by redefining values at the odd points.

**Conclusion:**

The two families of exact solutions obtained in this paper can be useful to study of related numerical methods and algorithms such as the finite difference method, the finite element method and the finite volume method that are applied by scientists to simulate the fluids for applications.

## Background and Main results

The *N*-dimensional isentropic compressible Euler equations with a damping term are written as

1 $$\begin{aligned} \left\{ \begin{array}{l} \displaystyle \rho _t+{\nabla }\cdot (\rho u)=0,\\ \displaystyle \rho \left[ u_t+(u\cdot {\nabla })u\right] +{\nabla }p+\alpha \rho u=0, \end{array}\right. \end{aligned}$$

where $\rho (t,x):[0,\infty )\times \mathbb {R}^N\rightarrow [0,\infty )$ and $u(t,x):[0,\infty )\times \mathbb {R}^N\rightarrow \mathbb {R}^N$ represent the density and the velocity of the fluid respectively. *p* represents the pressure function, which is given by

2 $$\begin{aligned} p=K\rho ^{\gamma },\quad K>0, \gamma \ge 1, \end{aligned}$$

by the adiabatic $\gamma$-law. The constant $\alpha \ge 0$ is the damping coefficient.

System (1) is one of the most fundamental equations in fluid dynamics. Many interesting fluid dynamic phenomena can be described by system (1) (Lions , 1998a; Lions , 1998b). The Euler equations $(\alpha =0)$ are also the special case of the noted Navier–Stokes equations, whose problem of whether there is a formation of singularity is still open and long-standing. Thus, the singularity formation in fluid mechanics has been attracting the attention of a number of researchers (Sideris 1985; Xin 1998; Suzuki 2013; Lei et al. 2013; Li and Wang 2006; Li et al. 2013).

Among others, we mention that in 2003, Sideris–Thomases–Wang (Sideris et al. 2003) obtained results for the three dimensional compressible Euler equations with a linear damping term with assumption $\gamma >1$, that is, system (1) with $N=3$ and $\gamma >1$. They discovered that damping prevents the formation of singularities in small amplitude flows, but large solutions may still break down. They formulated the Euler system as a symmetric hyperbolic system, established the finite speed of propagation of the solution, and some energy estimates to obtain local existence as well as global existence of the solution. For larger solution, they showed that the solution will blow up in a finite time by establishing certain differential inequalities.

In this article, we consider the one dimensional case of system (1):

3 $$\begin{aligned} \left\{ \begin{array}{l} \displaystyle \rho _t+\rho _x u+\rho u_x=0,\\ \displaystyle \rho (u_t+uu_x)+p_x+\alpha \rho u=0. \end{array}\right. \end{aligned}$$

More precisely, we apply the perturbational method to obtain the following main results.

### **Theorem 1**

*For system* (3) *with*$\gamma >1$*and*$\alpha >0$, *one has the following family of exact solutions with parameters*$\xi , \rho (0,0)>0, a_0>0$*and*$a_1$:

4 $$\left\{ {\begin{array}{*{20}l} {\rho (t,x) = \left[ {\max \left\{ {\rho ^{{\gamma - 1}} (t,0) - \frac{{\gamma - 1}}{{K\gamma }}\left[ {\left( {\frac{{\ddot{a}}}{a} + \alpha \frac{{\dot{a}}}{a}} \right)\frac{{x^{2} }}{2} + \left( {\dot{b} + b\frac{{\dot{a}}}{a} + \alpha b} \right)x} \right],0} \right\}} \right]^{{\frac{1}{{\gamma - 1}}}} ,} \\ {u(t,x) = \frac{{\dot{a}}}{a}x + b,} \\ \end{array} } \right.$$

*where*$\rho ^{\gamma -1}(t,0)$*is given by*

5 $$\begin{aligned} \displaystyle \rho ^{\gamma -1}(t,0)=\rho ^{\gamma -1}(0,0)e^{-\int _0^t(\gamma -1)\frac{\dot{a}}{a}ds}+\int _0^t\frac{\gamma -1}{K\gamma }b\left( \dot{b}+b\frac{\dot{a}}{a}+\alpha b\right) e^{\int _0^s(\gamma -1)\frac{\dot{a}}{a}dr}ds. \end{aligned}$$

*a*(*t*) *andb*(*t*) *satisfy the following ordinary differential equations:*

6 $$\left\{ {\begin{array}{*{20}l} {\ddot{a} + \alpha \dot{a} = \xi /a^{\gamma } ,} & {\xi \in \mathbb{R},} \\ {a(0) = a_{0} gt;0,} & {} \\ {\dot{a}(0) = a_{1} \in \mathbb{R},} & {} \\ \end{array} } \right.$$

7 $$\begin{aligned}&\left\{ \begin{array}{ll} {}\ddot{b}+f(t)\dot{b}+g(t)b=0,\\ {}f(t):=(\gamma +1)\frac{\dot{a}}{a}+\alpha ,\\ {}g(t):=2\frac{\ddot{a}}{a}+(\gamma -1)\frac{\dot{a}^2}{a^2}+(\gamma +1)\alpha \frac{\dot{a}}{a}. \end{array}\right. \end{aligned}$$

### *Remark 2*

The ordinary differential equation (O.D.E.) (6) will be analyzed in section 2 and it is well-known by the theory of ordinary differential equations that the solutions of system (7) exist and is $C^2$ as long as *f* and *g*, which are functions of $\ddot{a}$, $\dot{a}$ and *a*, are continuous.

### **Theorem 3**

*For the family of exact solutions in Theorem*1, *we have the following five cases.*

*(i)*: *If*$\xi >0$*and*$a_1\ge 0$, *then the solution* (4) *is a global solution.*
*(ii)*: *If*$\xi >0, a_1<0$*and*$a_0>-a_1/\alpha$, *then the solution* (4) *is a global solution.*
*(iii)*: *If*$\xi <0$, *then the solution* (4) *blows up on a finite time.*
*(iv)*: *If*$\xi =0$*and*$a_1>0$, *then the solution* (4) *blows up on the finite time*$T=\frac{1}{\alpha }\ln \frac{a_1}{a_1+a_0\alpha }>0$.
*(v)*: *If*$\xi =0$, *and*$a_1<0$*and*$a_0<-a_1/\alpha$, *then the solution* (4) *blows up on the finite time*$T=\frac{1}{\alpha }\ln \frac{a_1}{a_1+a_0\alpha }>0$.

Moreover, we show that the singularity formations in the cases *iii*), *iv*) and *v*) above are of essential type in the sense that the singularities cannot be smoothed by redefining values at the odd points. This is an improvement of the corresponding results in Yuen (2011).

For $\gamma =1$, we obtain the following theorem.

### **Theorem 4**

*For system* (3) *with*$\gamma =1$*and*$\alpha >0$, *one has the following family of exact solutions with parameters*$\xi , \rho (0,0)>0, a_0>0$*and*$a_1$.

8 $$\begin{aligned} \left\{ \begin{array}{l} \displaystyle \rho (t,x)=\rho (0,0)e^{h(t,x)},\\ \displaystyle u(t,x)=\frac{\dot{a}}{a}x+b, \end{array}\right. \end{aligned}$$

*where*

9 $$\begin{aligned} h(t,x):=\int _0^t\left[ \frac{b}{K}\left( \dot{b}+\alpha \frac{\dot{a}}{a}b+b\right) -\frac{\dot{a}}{a}\right] ds-\frac{1}{K}\left[ \frac{1}{2}\left( \frac{\ddot{a}}{a} +\alpha \right) x^2+\left( \dot{b}+\frac{\dot{a}}{a}b+\alpha b\right) x\right] . \end{aligned}$$

*a*(*t*) *andb*(*t*) *satisfy the following ordinary differential equations:*

10 $$\begin{aligned}&\left\{ \begin{array}{ll} {}\ddot{a}+\alpha \dot{a}=\xi /a,\quad \xi \in \mathbb {R},\\ {}a(0)=a_0>0,\\ {}\dot{a}(0)=a_1\in \mathbb {R}, \end{array}\right. \end{aligned}$$

11 $$\begin{aligned}&\left\{ \begin{array}{ll} {}\ddot{b}+f_1(t)\dot{b}+g_1(t)b=0,\\ {}f_1(t):=2\frac{\dot{a}}{a}+\alpha ,\\ {}g_1(t):=2\frac{\ddot{a}}{a}+2\alpha \frac{\dot{a}}{a}. \end{array}\right. \end{aligned}$$

### **Theorem 5**

*For the family of exact solutions in Theorem*4, *we have the following five cases.*

*(i)*: *If*$\xi >0$*and*$a_1\ge 0$, *then the solution* (8) *is a global solution.*
*(ii)*: *If*$\xi >0, a_1<0$*and*$a_0>-a_1/\alpha$, *then the solution* (8) *is a global solution.*
*(iii)*: *If*$\xi <0$, *then the solution* (8) *blows up on a finite time.*
*(iv)*: *If*$\xi =0$*and*$a_1>0$, *then the solution* (8) *blows up on the finite time*$T=\frac{1}{\alpha }\ln \frac{a_1}{a_1+a_0\alpha }>0$.
*(v)*: *If*$\xi =0$, *and*$a_1<0$*and*$a_0<-a_1/\alpha$, *then the solution* (8) *blows up on the finite time*$T=\frac{1}{\alpha }\ln \frac{a_1}{a_1+a_0\alpha }>0$.

## Analysis of an O.D.E.

Consider the following initial value problem.

12 $$\begin{aligned} \left\{ \begin{array}{c} \ddot{a}+\alpha \dot{a}=\xi /a^\gamma ,\\ a(0)=a_0>0,\\ \dot{a}(0)=a_1\in \mathbb {R}, \end{array}\right. \end{aligned}$$

where $\gamma \ge 1$, $\alpha >0$ and $\xi \in \mathbb {R}$ are constants. We set

13 $$\begin{aligned} T^*:=\sup \{T>0:a(t)>0\text { on }[0,T)\}>0. \end{aligned}$$

### **Lemma 6**

*For system* (12), *if*$T^*$*is finite, then the one-sided limit*

14 $$\begin{aligned} \lim _{t\rightarrow T^*}a(t)=0. \end{aligned}$$

### *Proof*

Note that we always have

15 $$\begin{aligned} \lim _{t\rightarrow T^*}a(t)\ge 0. \end{aligned}$$

Suppose $\displaystyle \lim _{t\rightarrow T^*}a(t)>0$. Then we can extend the solution of (12) to $[0, T^*+\varepsilon )$ by solving the following system.

16 $$\begin{aligned} \left\{ \begin{array}{l} \ddot{a}+\alpha \dot{a}=\xi /a^\gamma ,\\ a(T^*)=\displaystyle \lim _{t\rightarrow T^*}a(t)>0,\\ \dot{a}(T*)=\displaystyle \lim _{t\rightarrow T^*}\dot{a}(t)\in \mathbb {R}. \end{array}\right. \end{aligned}$$

This contradicts the definition of $T^*$. Thus, the lemma is established. $\square$

### **Lemma 7**

*For system* (12), *we have the following three cases.*

- *Case 1.If*$$\xi >0 \quad \text {and} \quad a_1\ge 0, \quad \text{then} \quad T^*=+\infty$$ .
- *Case 2.If*$$\xi >0, a_1<0 \quad \text {and} \quad a_0>-a_1/\alpha , \quad \text {then} \quad T^*=+\infty$$ .
- *Case 3.If*$$\xi <0, \quad \text {then} \quad T^*<+\infty$$.

### *Proof*

Suppose $\xi >0$ and $T^*<+\infty$. Then, for all $t\in [0,T^*)$, we have

17 $$\begin{aligned} \ddot{a}+\alpha \dot{a}&\ge 0, \end{aligned}$$

18 $$\begin{aligned} \frac{\mathrm {d} }{\mathrm {d} t}\left( e^{\alpha t}\dot{a}\right)&\ge 0,\end{aligned}$$

19 $$\begin{aligned} e^{\alpha t}\dot{a}&\ge a_1,\end{aligned}$$

20 $$\begin{aligned} \dot{a}&\ge a_1e^{-\alpha t},\end{aligned}$$

21 $$\begin{aligned} a&\ge a_0+\frac{a_1}{\alpha }\left( 1-\frac{1}{e^{\alpha t}}\right) . \end{aligned}$$

It follows that if $a_1\ge 0$, then $\displaystyle \lim _{t\rightarrow T^*}a(t)>0$, and if $a_1<0$ and $a_0>-a_1/\alpha$, then $\displaystyle \lim _{t\rightarrow T^*}a(t)>0$. This is impossible by Lemma 6. Thus, Case 1. and Case 2. of the lemma are established.

Now, suppose $\xi <0$. If $T^*=+\infty$, then for all $t>0$, we have

22 $$\begin{aligned} \ddot{a}+\alpha \dot{a}&\le 0. \end{aligned}$$

Reversing the inequalities from (17) to (21), we have

23 $$\begin{aligned} a&\le a_0+\frac{a_1}{\alpha }\left( 1-\frac{1}{e^{\alpha t}}\right) , \end{aligned}$$

24 $$\begin{aligned}&\le a_0+\frac{|a_1|}{\alpha }. \end{aligned}$$

Thus,

25 $$\begin{aligned} \ddot{a}+\alpha \dot{a}=\frac{\xi }{a^\gamma }\le \frac{\xi }{\left( a_0+|a_1|/\alpha \right) ^\gamma }=:A<0. \end{aligned}$$

Thus,

26 $$\begin{aligned} \ddot{a}+\alpha \dot{a}&\le A, \end{aligned}$$

27 $$\begin{aligned} a&\le a_0+\left( \frac{a_1}{\alpha }-\frac{A}{\alpha ^2}\right) \left( 1-\frac{1}{e^{\alpha t}}\right) +\frac{A}{\alpha }t,\end{aligned}$$

28 $$\begin{aligned} a&\le a_0+\left| \frac{a_1}{\alpha }-\frac{A}{\alpha ^2}\right| +\frac{A}{\alpha }t. \end{aligned}$$

As $A/\alpha <0$, we have $a(t)<0$ for all sufficiently large *t*. This is impossible as $T^*=+\infty$. Thus, Case 3. is established. $\square$

### *Remark 8*

The case for $\xi =0$ will be analyzed in the proof of Theorem 3.

## Proofs of the Theorems

### *Proof of Theorem 1*

We divide the proof into steps.

**Step 1.** In the first step, we show a lemma.

### **Lemma 9**

*For the* 1-*dimensional Euler equations with damping* (3) *with*$\gamma >1$*and*$\rho (0,0)>0$, *we have the following relation.*

29 $$\begin{aligned}&\frac{\partial }{\partial t}\left( \frac{\rho ^{\gamma -1}(t,0)}{\gamma -1}\right) +u_x\rho ^{\gamma -1}(t,0) -\frac{1}{K\gamma }\left( u_t+uu_x+\alpha u\right) u \nonumber \\&\quad -\frac{\gamma -1}{K\gamma }u_x\int _0^x\left( u_t+uu_x+\alpha u\right) (t,y)dy\nonumber \\&\quad -\frac{1}{K\gamma }\int _0^x\left( u_{tt}+uu_{xt}+u_xu_t+\alpha u_t\right) (t,y)dy=0. \end{aligned}$$

### *Proof of Lemma 9*

It is well known that $\rho$ is always positive if $\rho (0,0)$ is set to be positive. From (3)$_2$, we have, for $\rho (0,0)>0$,

30 $$\begin{aligned}&u_t+uu_x+\frac{1}{\rho }\frac{\partial }{\partial x}\left( K\rho ^\gamma \right) +\alpha u=0, \end{aligned}$$

31 $$\begin{aligned}&u_t+uu_x+\frac{K\gamma }{\gamma -1}\frac{\partial }{\partial x}\rho ^{\gamma -1}+\alpha u=0. \end{aligned}$$

Thus, we have

32 $$\begin{aligned} \frac{\partial }{\partial x}\frac{\rho ^{\gamma -1}}{\gamma -1}=-\frac{1}{K\gamma }\left( u_t+uu_x+\alpha u\right) . \end{aligned}$$

Taking integration with respect to *x*, we obtain

33 $$\begin{aligned} \frac{\rho ^{\gamma -1}(t,x)}{\gamma -1}=\frac{\rho ^{\gamma -1}(t,0)}{\gamma -1} -\frac{1}{K\gamma }\int _0^x\left( u_t+uu_x+\alpha u\right) (t,y)dy. \end{aligned}$$

On the other hand, multiplying $\rho ^{\gamma -2}$ on both sides of (3)$_1$, we get

34 $$\begin{aligned} \frac{\partial }{\partial t}\frac{\rho ^{\gamma -1}(t,x)}{\gamma -1}+u\frac{\partial }{\partial x}\frac{\rho ^{\gamma -1}(t,x)}{\gamma -1}+\rho ^{\gamma -1}u_x=0. \end{aligned}$$

From (33), we have

35 $$\begin{aligned} \rho ^{\gamma -1}=\rho ^{\gamma -1}(t,0)-\frac{\gamma -1}{K\gamma }\int _0^x\left( u_t+uu_x +\alpha u\right) (t,y)dy, \end{aligned}$$

and

36 $$\begin{aligned} \frac{\partial }{\partial t}\frac{\rho ^{\gamma -1}(t,x)}{\gamma -1}=\frac{\partial }{\partial t}\frac{\rho ^{\gamma -1}(t,0)}{\gamma -1}-\frac{1}{K\gamma }\int _0^x\left( u_{tt} +u_tu_x+uu_{xt}+\alpha u_t\right) (t,y)dy. \end{aligned}$$

Substituting (36), (32) and (35) into (34), one obtains the relation claimed in the lemma. $\square$

**Step 2.** We set

37 $$\begin{aligned} u=cx+b, \end{aligned}$$

where $c:=c(t)$ and $b:=b(t)$ are functions of *t*. Then, (29) is transformed to

38 $$\begin{aligned}&-\frac{1}{2K\gamma }\left\{ \frac{\mathrm {d} }{\mathrm {d} t}\left( \dot{c}+c^2\right) +\alpha (\dot{c}+c^2)+(\gamma +1)c(\dot{c}+c^2)+\gamma \alpha c^2\right\} x^2\nonumber \\&-\frac{1}{K\gamma }\left\{ \ddot{b}+\left[ (\gamma +1)c+\alpha \right] \dot{b}+\left[ 2\dot{c}+(\gamma +1)c^2+(\gamma +1)\alpha c\right] b\right\} x+\nonumber \\&\frac{1}{\gamma -1}\left\{ \frac{\partial }{\partial t}\rho ^{\gamma -1}(t,0)+(\gamma -1)c\rho ^{\gamma -1}(t,0) -\frac{\gamma -1}{K\gamma }b\left( \dot{b}+bc+\alpha b\right) \right\} =0, \end{aligned}$$

where we arrange the terms according to the coefficients of *x*.

**Step 3.** We use the Hubble transformation:

39 $$\begin{aligned} c=\frac{\dot{a}}{a}, \end{aligned}$$

and set the coefficient of (38) to be zero. Thus,

40 $$\begin{aligned} \frac{\dddot{a}}{a}+\alpha \frac{\ddot{a}}{a}+\gamma \frac{\dot{a} \ddot{a}}{a^2}+\gamma \alpha \frac{\dot{a}^2}{a^2}=0. \end{aligned}$$

Note that we have the novel identity

41 $$\begin{aligned} \dot{c}+c^2=\frac{\ddot{a}}{a}. \end{aligned}$$

Multiplying the both sides of (40) by $a^{\gamma +1}$, it becomes

42 $$\begin{aligned}&\frac{\mathrm {d} }{\mathrm {d} t}\left[ a^\gamma (\ddot{a} +\alpha \dot{a})\right] =0, \end{aligned}$$

43 $$\begin{aligned}&a^\gamma (\ddot{a}+\alpha \dot{a})=\xi , \end{aligned}$$

for some constant $\xi$.

**Step 4.** With (39), we set the coefficient of *x* in (38) to be zero. Thus, *b* satisfies

44 $$\begin{aligned} \ddot{b}+f(t)\dot{b}+g(t)b=0, \end{aligned}$$

where

45 $$\begin{aligned}&f(t):=(\gamma +1)\frac{\dot{a}}{a}+\alpha , \end{aligned}$$

46 $$\begin{aligned}&g(t):=2\frac{\ddot{a}}{a}+(\gamma -1)\frac{\dot{a}^2}{a^2}+(\gamma +1)\alpha \frac{\dot{a}}{a}. \end{aligned}$$

**Last Step.** With (39) and setting the coefficient of 1 in (38) to be zero, we are required to solve

47 $$\begin{aligned} {F}'(t)+G(t)F(t)=H(t), \end{aligned}$$

where

48 $$\begin{aligned}&F(t):=\rho ^{\gamma -1}(t,0), \end{aligned}$$

49 $$\begin{aligned}&G(t):=(\gamma -1)\frac{\dot{a}}{a},\end{aligned}$$

50 $$\begin{aligned}&H(t):=\frac{\gamma -1}{K\gamma }b\left( \dot{b}+b\frac{\dot{a}}{a}+\alpha b\right) . \end{aligned}$$

Solving the O.D.E (47) by the method of integral factor, one arrives at the solutions. The proof of Theorem 1 is complete. $\square$

Next, we prove Theorem 3 as follows.

### *Proof of Theorem 3*

For $\xi >0$, case *i*) and case *ii*) of Theorem 3 follow from Case 1. and Case 2. of Lemma 7.

For $\xi <0$, by Case 3. of Lemmas 6 and7, there exists a finite $T^*>0$ such that the one-sided limit of *a*(*t*) is zero as *t* approaches to $T^*$. It remains to show $T^*$ is not a removable singularity of $\dot{a}/a$. To this end, suppose one has

51 $$\begin{aligned} \lim _{t\rightarrow T^*}\dot{a}(t)=0. \end{aligned}$$

Then,

52 $$\begin{aligned} \lim _{t\rightarrow T^*}\frac{\dot{a}}{a}=\lim _{t\rightarrow T^*}\frac{\ddot{a}}{\dot{a}}=\lim _{t\rightarrow T^*}\frac{\xi /a^\gamma -\alpha \dot{a}}{\dot{a}}=-\infty . \end{aligned}$$

Thus, the singularity is of essential type and case *iii*) of Theorem 3 is proved.

For $$\xi =0$$, (6)$$_1$$ becomes

53 $$\begin{aligned} \ddot{a}+\alpha \dot{a}=0, \end{aligned}$$

which can be solved by using integral factor. The solution is

54 $$\begin{aligned} a(t)=a_0+\frac{a_1}{\alpha }\left( 1-\frac{1}{e^{\alpha t}}\right) . \end{aligned}$$

Thus, $a(T)=0$ if $a_1>0$. Also, $a(T)=0$ if $a_1<0$ and $a_0<-a_1/\alpha$, where $T:=\frac{1}{\alpha }\ln \frac{a_1}{a_1+a_0\alpha }>0$. As

55 $$\begin{aligned} \dot{a}(T)=a_1+\alpha a_0\ne 0, \end{aligned}$$

(*T*, *x*) is an essential singularity of *u*(*t*, *x*) for any *x*. Thus, cases *iv*) and *v*) of Theorem 3 are established. The proof is complete. $\square$

### *Proof of Theorems 4 and 5*

The corresponding relation of Lemma 9 for $\gamma =1$ is

56 $$\begin{aligned} \frac{\mathrm {d}}{\mathrm {d}t}\ln \rho (t,0)+u_x-\frac{1}{K}[u_t+uu_x+\alpha u]u-\frac{1}{K}\int _0^x[u_{tt}+u u_{xt}+u_tu_x+\alpha u_t](t,y)dy=0. \end{aligned}$$

With similar steps, one can obtain the family of exact solutions in Theorem 4.

Note that (10) is a special case of (12) and the arguments in the proof of Theorem 3 hold for $\gamma =1$. Thus, the results for Theorem 5 follows. $\square$

## Conclusion

The complicated Euler equations with a damping term (1) do not have a general solution in a closed form for arbitrary well-posed initial value problems. Thus, numerical methods and algorithms such as the finite difference method, the finite element method and the finite volume method are applied by scientists to simulate the fluids for applications in real world. Thus, our exact solutions in this article provide concrete examples for researchers to test their numerical methods and algorithms.
